# Beyond the Obvious: Modifiable Patient, Behavioral, and System Factors That Influence Postoperative Recovery in Surgery

**DOI:** 10.7759/cureus.111037

**Published:** 2026-06-17

**Authors:** John Salib, Mark Salib, Frederick Tiesenga

**Affiliations:** 1 School of Medicine, St. George's University School of Medicine, St. George's, GRD; 2 Department of General Surgery, West Suburban Medical Center, Chicago, USA

**Keywords:** enhanced recovery after surgery (eras), health optimization, modifiable factors, patient-centered care, postoperative recovery, prehabilitation, quality improvement, risk factors, surgical outcomes, surgical recovery pathways

## Abstract

Postoperative recovery represents a complex, multidimensional process that extends beyond the technical success of surgery, encompassing the restoration of physiologic stability, functional capacity, and patient-centered well-being. Recovery trajectories are highly variable, influenced not only by baseline patient characteristics but also by modifiable determinants spanning physiologic, behavioral, and system-level domains. This narrative review synthesizes current evidence on the interplay among these factors, highlighting mechanistic pathways, contextual modulators, and opportunities for targeted optimization. At the patient level, physiologic reserve, including nutritional status, sarcopenia, frailty, metabolic control, and comorbidities, defines the capacity to tolerate surgical stress and engage in functional recovery. Behavioral determinants, encompassing early mobilization, structured rehabilitation, patient engagement, perioperative education, and multimodal pain management, translate physiologic potential into meaningful postoperative outcomes. These factors operate synergistically, with behavioral interventions mitigating vulnerabilities such as frailty or sarcopenia while amplifying gains from physiologic optimization. System-level determinants, including multidisciplinary perioperative pathways, coordinated surgical home models, telemonitoring platforms, and implementation science-driven frameworks, provide the structural scaffolding necessary to ensure fidelity, adaptability, and scalability of interventions. Digital health technologies and predictive analytics offer additional mechanisms to monitor recovery trajectories, identify deviations early, and personalize care in real time. The integration of patient-level, behavioral, and system-level determinants is nonlinear and context-dependent, with interactions modulated by institutional resources, patient risk profiles, and care culture. Effective perioperative optimization requires a holistic, reproducible framework that aligns physiologic enhancement, behavioral engagement, and system-level support, enabling dynamic adaptation to individual patient needs. This review emphasizes that recovery is not a passive outcome but a mechanistically coherent, modifiable, and measurable process. By conceptualizing postoperative recovery as an integrated, adaptive, and precision-guided continuum, clinicians and health systems can improve functional outcomes, reduce complications, and advance patient-centered surgical care across heterogeneous populations.

## Introduction and background

Postoperative recovery is a central determinant of surgical outcomes and encompasses a multidimensional process that extends beyond the immediate technical success of an operation [1-3]. In addition to the absence of major complications, recovery involves the restoration of physiologic stability, functional capacity, and patient-reported well-being [1,3]. Variability in postoperative recovery trajectories has important implications for both individual patient outcomes and broader healthcare system performance, influencing complication rates, hospital length of stay (LOS), readmissions, and overall resource utilization [1,2,4]. Despite substantial advances in surgical technique, anesthesia, and perioperative management, significant heterogeneity in recovery persists across surgical populations, suggesting that factors beyond procedural complexity alone contribute to postoperative outcomes [1-3,5]. Although many perioperative optimization strategies have been developed and studied predominantly in elective surgical populations, postoperative recovery is equally important in emergency general surgery, where limited opportunities for preoperative optimization, greater physiologic derangement, and increased illness severity may influence recovery trajectories and the effectiveness of modifiable interventions.

Historically, perioperative risk stratification has focused primarily on relatively fixed patient characteristics such as age, baseline comorbidities, and operative risk [6,7]. While these factors remain important predictors of postoperative morbidity and mortality, growing evidence suggests that recovery is also shaped by a range of modifiable determinants that operate across the perioperative continuum [8-10]. These determinants include patient-level physiologic factors such as nutritional status, frailty, and physical conditioning [10-16]; behavioral factors, including patient engagement, early mobilization, and adherence to postoperative care plans [17-20]; and system-level influences, including care delivery models, standardized recovery pathways, and multidisciplinary coordination [1,21]. Recognizing the interplay between these domains has become increasingly relevant as surgical care evolves toward more integrated and patient-centered models [21].

Among system-level strategies, Enhanced Recovery After Surgery (ERAS) pathways have emerged as one of the most widely studied frameworks for optimizing perioperative care [1-3,5,22]. ERAS protocols integrate multiple evidence-based interventions, including standardized analgesic regimens, early mobilization, nutritional support, and coordinated perioperative management, designed to attenuate the physiologic stress response to surgery and facilitate more rapid recovery [1-3,5,22,23]. A substantial body of evidence has associated ERAS implementation with reduced postoperative complications, shorter hospital stays, and lower healthcare costs across multiple surgical specialties [1-5,22]. However, outcomes may vary according to procedural context, patient characteristics, and adherence to protocol components, underscoring the importance of understanding how individual perioperative factors contribute to recovery trajectories [2-5].

Parallel to system-level initiatives, increasing attention has been directed toward preoperative optimization strategies to enhance physiologic reserve prior to surgery [8-10]. Prehabilitation programs, which often combine exercise training, nutritional support, and psychological preparation, have been proposed to improve functional capacity and resilience before the physiologic stress of surgery [8-12]. Several systematic reviews and meta-analyses suggest that multimodal prehabilitation interventions may be associated with improved postoperative outcomes in selected populations, particularly among patients undergoing major abdominal procedures [8-10]. Nevertheless, considerable heterogeneity exists across studies in intervention design, patient selection, and outcome measurement, underscoring the need for cautious interpretation and continued investigation in this area [8-12].

In addition to preoperative conditioning, patient-specific physiologic vulnerabilities such as frailty, sarcopenia, and malnutrition have increasingly been recognized as important determinants of postoperative recovery [6,7,13-16]. Frailty, a syndrome characterized by diminished physiologic reserve and increased vulnerability to stressors, has been associated with higher rates of postoperative complications, prolonged hospitalization, and increased mortality in surgical patients [6,7,13]. Similarly, sarcopenia and impaired nutritional status have been linked to adverse postoperative outcomes, including delayed recovery and increased morbidity following major abdominal surgery [14-16]. These findings highlight the potential value of identifying modifiable physiologic deficits during the preoperative period and incorporating targeted interventions into perioperative care pathways [10-12,14-16].

Beyond physiologic factors, behavioral determinants of recovery represent an additional dimension that may influence postoperative outcomes [17-20]. Patient engagement in recovery protocols, including early mobilization, adherence to postoperative rehabilitation strategies, and active involvement in perioperative education, has been associated with improved functional recovery and reduced complication rates in several clinical contexts [17-20,24,25]. Early postoperative mobilization, for example, has been shown to improve oxygenation, enhance functional capacity, and reduce postoperative complications following major abdominal surgery [17-20]. Similarly, structured patient education and perioperative counseling interventions may help improve patient preparedness, adherence to recovery protocols, and postoperative outcomes [24,25]. Effective pain management strategies, particularly those employing multimodal analgesic approaches, also play an important role in facilitating early mobilization and functional recovery while minimizing reliance on opioid-based therapies [23].

These behavioral and physiologic factors operate within broader healthcare system frameworks that influence how perioperative care is delivered [21]. Multidisciplinary care models, such as the perioperative surgical home, aim to coordinate care across the preoperative, intraoperative, and postoperative phases through structured collaboration among surgeons, anesthesiologists, nurses, and allied health professionals [21]. Such models aim to reduce care fragmentation, improve communication among clinical teams, and facilitate the implementation of standardized recovery protocols [21]. Increasing attention has therefore been directed toward understanding how organizational and system-level factors interact with patient and behavioral determinants to shape postoperative recovery trajectories [21].

More recently, technological innovations have expanded opportunities to monitor and optimize postoperative recovery. Wearable physiologic sensors, mobile health platforms, and remote monitoring systems may provide objective measures of activity and recovery outside the inpatient setting. At the same time, emerging applications of artificial intelligence (AI) and predictive analytics have been proposed to improve risk stratification and guide individualized perioperative management [26-30]. Although these approaches remain under active investigation, they represent promising tools for advancing data-driven and patient-centered perioperative care [26-30].

Taken together, the existing literature suggests that postoperative recovery is influenced by a complex interplay of patient-level physiologic factors, including frailty, nutritional status, and physical conditioning; behavioral determinants, such as patient engagement and early mobilization; and system-level characteristics of care delivery, including enhanced recovery pathways and multidisciplinary perioperative care models. Although substantial progress has been made in identifying individual contributors to postoperative outcomes, the relationships among these domains remain incompletely characterized, and the relative impact of modifiable factors across different surgical populations remains an area of active study.

The aim of this narrative review is therefore to synthesize the current evidence regarding modifiable determinants of postoperative recovery in general surgery, with particular focus on patient-level physiologic factors, behavioral determinants of recovery, and system-level models of perioperative care. By examining these domains collectively, this review seeks to provide a structured overview of factors that may influence recovery trajectories, while highlighting areas where further research could refine perioperative strategies to improve surgical outcomes.

History

Understanding postoperative recovery has undergone substantial evolution over the past several decades as surgical practice has shifted from a procedure-focused paradigm toward a more comprehensive model of perioperative care [1,2]. Historically, the success of surgical interventions was evaluated primarily through immediate technical outcomes, including intraoperative complications, short-term mortality, and procedural completion [1]. Postoperative management frequently emphasized prolonged fasting, delayed ambulation, liberal opioid analgesia, and extended inpatient observation [1,23]. Although intended to minimize perioperative risk, these practices often contributed to prolonged hospitalization, delayed functional recovery, and increased exposure to hospital-associated complications [1,2,23].

During the latter half of the 20th century, advances in surgical physiology led to greater recognition of the systemic responses that occur following operative intervention [1,5]. Surgical trauma initiates a coordinated neuroendocrine, metabolic, and inflammatory response characterized by activation of the hypothalamic-pituitary-adrenal axis, increased sympathetic activity, insulin resistance, and accelerated protein catabolism [1,5]. While these responses are adaptive following acute injury, excessive or prolonged activation may contribute to impaired immune function, delayed gastrointestinal recovery, metabolic dysregulation, and reduced functional capacity [1,3-5]. The growing appreciation of the surgical stress response prompted efforts to develop perioperative strategies to attenuate physiologic stress and facilitate earlier recovery [1,5].

One of the most influential developments arising from this shift was the introduction of structured multimodal perioperative care pathways, later formalized as Enhanced Recovery After Surgery (ERAS) programs [1-4,22]. Initially developed within colorectal surgery, ERAS integrated evidence-based interventions such as optimized analgesia, early enteral nutrition, early mobilization, and coordinated multidisciplinary care to reduce surgical stress and accelerate recovery [1-4,21-23]. Subsequent adoption across numerous surgical specialties was associated with improvements in recovery-related outcomes, including reduced complications and shorter hospital stays [1-4,22,24]. The success of ERAS helped establish the concept that postoperative recovery is influenced not only by the technical conduct of an operation but also by the broader perioperative environment in which care is delivered [1-4,21,22].

This evolving perspective has expanded the focus of perioperative care beyond traditional measures of operative risk toward a more comprehensive understanding of recovery. Contemporary approaches increasingly recognize postoperative recovery as a multidimensional process shaped by interactions among physiologic reserve, patient behaviors, perioperative interventions, and healthcare system factors. These developments have provided the foundation for ongoing efforts to identify modifiable determinants of recovery and optimize outcomes across diverse surgical populations.

## Review

Methods

This qualitative narrative review was undertaken to comprehensively examine modifiable patient, behavioral, and system factors that influence postoperative recovery in general surgery. Recognizing the breadth and heterogeneity of the available literature, we adopted an adaptive framework informed by the Preferred Reporting Items for Systematic Reviews and Meta-Analyses (PRISMA) guidelines. While this review does not adhere to a strictly systematic review methodology, we implemented key PRISMA principles, including clearly defined search strategies, explicit eligibility criteria, independent multi-reviewer screening, and structured data synthesis, to maximize methodological rigor, transparency, and reproducibility. The adaptive approach was selected to enable a detailed thematic and qualitative exploration of recovery-related factors across diverse surgical populations and study designs, while maintaining reproducibility and minimizing bias.

**Figure 1:**
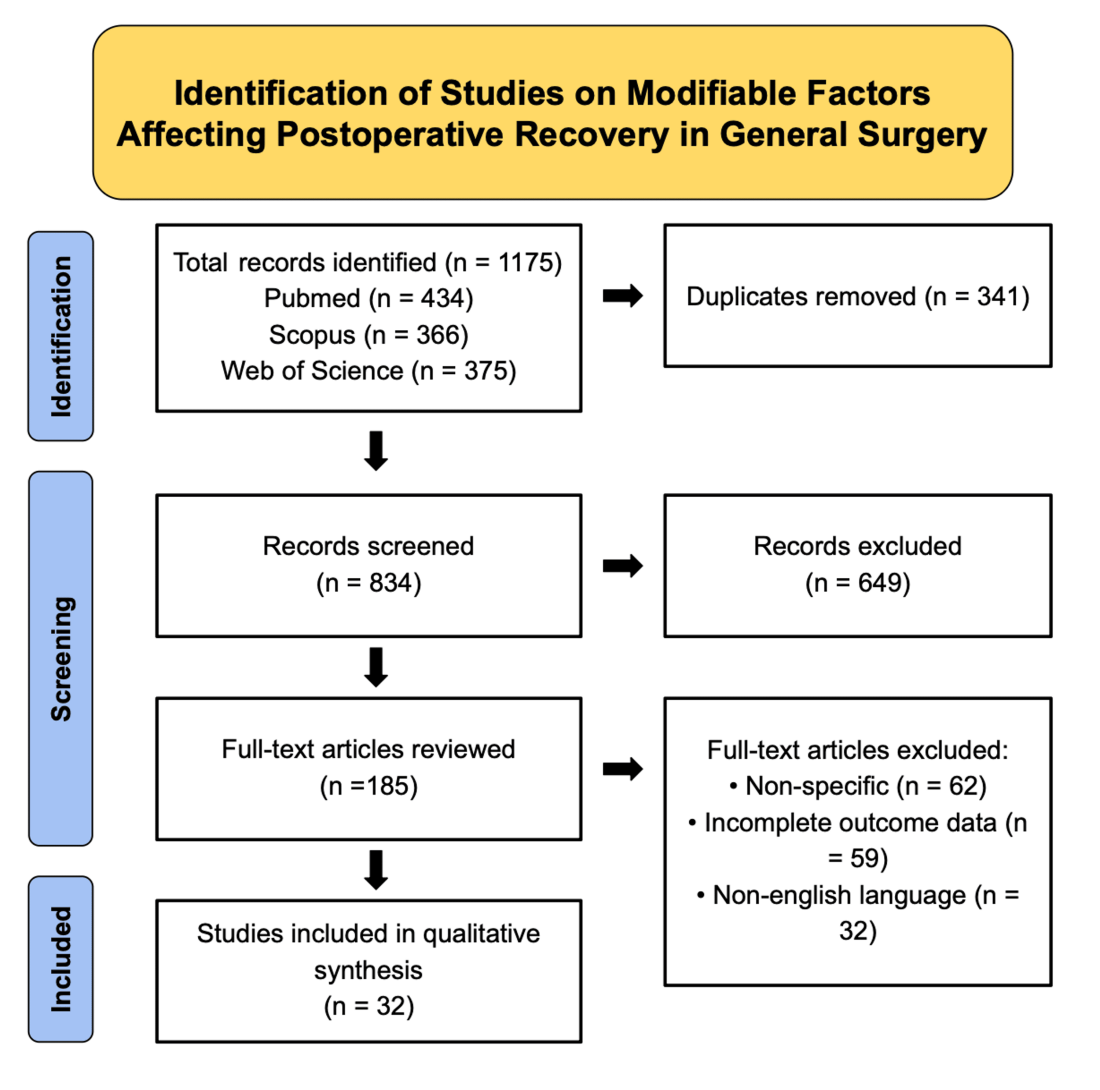
PRISMA Flow Diagram of Study Selection on Modifiable Factors Affecting Postoperative Recovery in Surgery Flowchart illustrating the systematic identification, screening, eligibility, and inclusion process for studies evaluating modifiable factors affecting postoperative recovery in surgery. A total of 1,175 records were identified through database searching, including PubMed (n = 434), Scopus (n = 366), and Web of Science (n = 375). After removal of 341 duplicates, 834 records were screened by title and abstract, of which 649 were excluded. A total of 185 full-text articles were assessed for eligibility, and 153 were excluded due to non-specific relevance (n = 62), incomplete outcome data (n = 59), or articles that are non-English (n = 32). A total of 32 studies were included in the final qualitative synthesis. PRISMA: Preferred Reporting Items for Systematic Reviews and Meta-Analyses

A comprehensive literature search (Figure 1) was conducted across PubMed (n = 434), Scopus (n = 366), and Web of Science (n = 375). Search strategies were developed iteratively by all authors in consultation, employing a combination of controlled vocabulary terms, keywords, and Boolean operators to maximize both sensitivity and specificity. Search terms included combinations of concepts related to Enhanced Recovery After Surgery (ERAS), prehabilitation, patient education, early mobilization, nutrition, frailty, sarcopenia, psychological well-being, wearable monitoring, and perioperative care pathways. In PubMed, the search strategy was structured using controlled vocabulary (MeSH terms where applicable), keywords, and Boolean operators as follows: (("enhanced recovery after surgery" OR "ERAS" OR "fast track surgery" OR "perioperative care pathway*") OR ("prehabilitation" OR "preoperative rehabilitation" OR "exercise training") OR ("early mobilization" OR "early ambulation" OR "postoperative mobilization") OR ("patient education" OR "perioperative education" OR "patient engagement") OR ("nutrition" OR "malnutrition" OR "nutritional support") OR ("frailty" OR "sarcopenia" OR "muscle weakness"[MeSH Terms]) OR ("psychological well-being" OR "anxiety" OR "depression" OR "psychological factors") OR ("wearable devices" OR "remote monitoring" OR "mobile health" OR "digital health") OR ("perioperative care" OR "perioperative medicine" OR "surgical recovery")) AND ("postoperative recovery" OR "postoperative outcomes" OR "surgical outcomes"). Equivalent search strategies were adapted for Scopus and Web of Science. To ensure comprehensiveness, reference lists of included studies and relevant reviews were also manually screened for additional eligible articles. To ensure comprehensiveness, we also screened references of included articles and relevant reviews to identify additional eligible studies. Duplicates across databases were systematically removed prior to title and abstract screening to ensure an accurate pool of unique studies.

Eligibility criteria were designed to capture studies that addressed modifiable factors influencing postoperative recovery in adult surgical populations. Included studies comprised randomized controlled trials (RCTs), prospective and retrospective observational studies, systematic reviews, meta-analyses, and narrative reviews. Studies were considered relevant if they examined patient-level factors (e.g., nutrition, physical frailty, sarcopenia, and psychological resilience), behavioral interventions (e.g., prehabilitation programs, early mobilization, and structured patient education), or system-level factors (e.g., ERAS protocols, perioperative care coordination, and monitoring technologies) associated with recovery outcomes. Only full-text articles published in English were included to ensure a thorough assessment of methodology and results. Studies were excluded if they focused on non-human subjects and non-surgical populations, or lacked primary outcome data pertinent to postoperative recovery. Additionally, case reports, conference abstracts, editorials, or opinion pieces without substantive data were excluded, as were redundant publications of the same patient cohort that did not provide unique or additional findings. Non-English studies were only included if key outcome data were available in English-language abstracts. These criteria were designed to maximize the relevance, quality, and interpretability of the included literature while reducing potential bias.

Three independent reviewers conducted all stages of screening and data extraction. Discrepancies in data extraction were resolved through discussion and consensus, with adjudication by a senior author when required. Initial title and abstract screening was followed by full-text review to determine eligibility, with discrepancies resolved through discussion and consensus among the reviewers. Prior to full extraction, the review team piloted a standardized data extraction form to ensure consistency, which captured study characteristics, patient demographics, surgical procedure type, intervention details, outcome measures, and key findings relevant to postoperative recovery. This structured approach ensured uniformity in data collection and minimized subjectivity in interpreting heterogeneous study designs and outcomes. The extracted data were compiled and are summarized in Table 1.

**Table 1:** Summary of Key Evidence on Modifiable Patient, Behavioral, and System Factors Influencing Postoperative Recovery in Surgery This table summarizes 32 studies on modifiable patient, behavioral, and system factors influencing postoperative recovery, including ERAS protocols, prehabilitation, frailty, nutrition, early mobilization, preoperative education, multimodal analgesia, and wearable/AI monitoring. Study designs range from systematic reviews and meta-analyses (level I) to RCTs and high-quality cohort studies (level II). Columns present study characteristics, populations, interventions, comparators, outcomes, and main findings, emphasizing factors that can optimize recovery [1-32]. ERAS: Enhanced Recovery After Surgery, AI: artificial intelligence, RCT: randomized controlled trial, LOS: length of stay, GI: gastrointestinal, CRS-HIPEC: cytoreductive surgery with hyperthermic intraperitoneal chemotherapy, RR: relative risk, CT: computed tomography, HR: hazard ratio, OR: odds ratio, 6MWT: 6-minute walk test

#	Reference (year)	Study design/level of evidence	Population/sample	Intervention/factor studied	Comparator/control	Outcomes measured	Main findings/effect
1	Lau and Chamberlain (2016) [1]	Meta-analysis, level I evidence	42 studies; 5,241 surgical patients (varied surgeries)	ERAS programs, including early mobilization, multimodal analgesia, early feeding, and reduced fasting	Conventional perioperative care	LOS, postoperative complications, mortality, readmission, GI recovery, costs	ERAS implementation significantly reduced LOS (~2.35 days), decreased overall complications (~38%), improved GI recovery, and reduced costs; no significant change in mortality or 30-day readmission. Demonstrates system-level interventions (care pathways) substantially modulate recovery outcomes.
2	Kannan et al. (2025) [2]	Systematic review, level I evidence	11 RCTs; 1,476 colorectal surgery patients	ERAS protocols, including standardized perioperative care bundles, multimodal analgesia, early feeding, and mobilization	Traditional perioperative care	LOS, complication rates, bowel function recovery, opioid use, and nutritional markers	ERAS associated with shorter LOS (3-8 days), lower postoperative complication rates, faster bowel recovery, decreased opioid requirements, and improved nutritional intake. Confirms reproducible benefit of structured perioperative protocols on multiple recovery domains.
3	Katikam et al. (2025) [3]	Prospective RCT, level II evidence	150 abdominal surgery patients	ERAS pathway: preoperative counseling, carb loading, early mobilization, opioid-sparing analgesia, and early diet	Standard care	LOS, time to bowel movement, complication incidence, and patient satisfaction	ERAS reduced LOS (4.6 versus 7.9 days), accelerated bowel function recovery (2.3 versus 4.1 days), decreased complications (10% versus 28%), and improved patient-reported satisfaction. Highlights both behavioral (mobilization, feeding) and system (protocolized care) factors impacting recovery.
4	Yue et al. (2024) [4]	Prospective cohort, level II evidence	136 patients undergoing CRS-HIPEC	ERAS-focused pain management: multimodal analgesia, opioid-sparing regimens, and early mobilization	Pre-ERAS pain care	Pain scores, opioid consumption, time to ambulation, diet initiation, and bowel function	ERAS reduced pain scores and opioid use, enabled earlier diet initiation, and promoted faster bowel recovery. Pain management and structured mobilization identified as key modifiable factors affecting recovery trajectory in high-risk surgery.
5	Bisch et al. (2021) [5]	Systematic review and meta-analysis, level I evidence	31 studies; 6,703 gynecologic oncology patients	ERAS protocols, including preoperative counseling, multimodal analgesia, and early diet/mobilization	Conventional perioperative care	LOS, complication rates, readmission, and cost	ERAS reduced LOS by ~1.6 days, complications by ~32%, readmissions by ~20%, and per-patient costs by ~$2129. Confirms system-wide protocol standardization enhances outcomes across specialty-specific surgical populations.
6	Steffens et al. (2024) [6]	Systematic review of RCTs, level I evidence	23 RCTs; 2,475 colorectal cancer surgery patients	Prehabilitation modalities: structured exercise, nutritional supplementation, and psychological preparation	Usual care	Complication rates, LOS, and functional recovery metrics	Prehabilitation improved postoperative functional outcomes and reduced infectious complications (RR: 0.65); modest LOS reductions (~0.87 days). Patient-level preoperative optimization (behavioral and physiologic) is critical in modulating recovery.
7	Widanage et al. (2025) [7]	Systematic review and meta-analysis, level I evidence	Multimodal prehabilitation in colorectal surgery patients	Combined prehabilitation: exercise, nutrition, and psychosocial support	Standard care	LOS, postoperative complications, and functional recovery	Multimodal prehabilitation improved physical function and reduced complications. Suggests synergistic benefits of targeting multiple modifiable patient-level factors concurrently.
8	Shen et al. (2024) [8]	Systematic review and meta-analysis, level I evidence	Patients undergoing esophagogastric cancer surgery	Nutrition-based prehabilitation: protein supplementation, immunonutrition, and caloric optimization	Standard care	LOS, morbidity, and postoperative complications	Preoperative nutritional interventions shortened LOS and decreased complication rates. Highlights nutrition as a high-impact modifiable patient factor.
9	Fradley et al. (2026) [9]	Narrative review, level V evidence	Abdominal surgery populations	Nutritional prehabilitation strategies: high-protein diet, immunonutrition, and preoperative counseling	Standard care/variable	Nutritional status, complication rates, and functional recovery	Nutritional optimization improves postoperative recovery, supports functional restoration, and reduces complications. Evidence supports integrating nutrition into prehabilitation as standard care.
10	Gillis et al. (2022) [10]	Narrative review, level V evidence	General surgical populations	Prehabilitation, ERAS, or combination	Standard care	LOS, functional recovery, and complication rates	Both ERAS and prehabilitation independently enhance recovery; combination strategies demonstrate additive benefits. Highlights integration of patient-level (exercise and nutrition) and system-level (protocolized care) factors for maximal recovery gains.
11	Oakland et al. (2016) [11]	Systematic review and meta-analysis, level I evidence	33 studies; 6,100+ surgical patients (various procedures)	Preoperative frailty assessed via validated indices (e.g., Fried, Rockwood)	Non-frail patients	Postoperative complications, mortality, LOS, and readmission	Frailty strongly associated with increased postoperative complications (RR: ~1.78), higher mortality, longer LOS, and increased readmissions. Highlights frailty as a modifiable patient-level factor through prehabilitation and optimization.
12	Shinall et al. (2020) [12]	Retrospective cohort, level II evidence	45,000+ adult surgical patients	Preoperative frailty (Risk Analysis Index) stratified by operative stress	Non-frail patients	30-day and 1-year postoperative mortality	Both frailty and high operative stress independently predicted higher short- and long-term mortality. Suggests targeted preoperative interventions for frail patients may reduce mortality risk.
13	Nguyenhuy et al. (2022) [13]	Systematic review and meta-analysis, level I evidence	12 studies; 3,500+ cardiac surgery patients	Fried Frailty Phenotype (weight loss, weakness, exhaustion, low activity, and slowness)	Non-frail patients	Postoperative morbidity, LOS, and mortality	Frailty increased the risk of postoperative morbidity (OR: 2.1) and mortality (OR: 2.7), prolonged LOS, and ICU stays. Supports routine frailty assessment as a modifiable preoperative strategy for risk stratification.
14	Weimann et al. (2017) [14]	ESPEN clinical guideline, level I evidence	Broad surgical populations	Preoperative and perioperative nutritional optimization: caloric/protein supplementation, immunonutrition, and enteral feeding	Standard nutritional care	Complications, LOS, wound healing, and functional recovery	Nutritional interventions reduce postoperative complications, improve wound healing, decrease LOS, and enhance functional recovery. Nutrition is a high-impact, modifiable patient factor.
15	Huang et al. (2015) [15]	Prospective cohort, level II evidence	154 colorectal cancer surgery patients	Sarcopenia measured via CT-based muscle mass, handgrip strength, and physical performance	Non-sarcopenic patients	Postoperative complications, LOS, and mortality	Sarcopenia independently predicted higher postoperative complications (HR: 2.3), delayed recovery, and prolonged LOS. Supports prehabilitation targeting muscle mass and functional capacity.
16	Nakanishi et al. (2018) [16]	Prospective cohort, level II evidence	180 colorectal cancer surgery patients	Sarcopenia assessment via skeletal muscle index	Non-sarcopenic patients	Postoperative complications and LOS	Sarcopenia predicted complications (OR: 3.1) and longer LOS. Highlights modifiable patient factors (nutrition and exercise) for improving outcomes.
17	Tariq and Ammar (2026) [17]	Cross-sectional study, level III evidence	212 bariatric surgery patients (sleeve gastrectomy versus gastric bypass)	Psychological and emotional status post-surgery	Comparative procedure analysis	Anxiety, depression, quality of life, and patient satisfaction	Sleeve gastrectomy associated with slightly better psychological outcomes; overall, emotional support and counseling modulate recovery trajectory, emphasizing behavioral interventions.
18	Svensson-Raskh et al. (2021) [18]	Single-center RCT, level II evidence	60 major abdominal surgery patients	Early mobilization (within 2 hours post-surgery)	Standard mobilization (24+ hours)	Oxygenation, peripheral perfusion, LOS, and complications	Early mobilization improved arterial oxygenation, perfusion, and functional recovery; reduced postoperative pulmonary complications. Demonstrates behavioral intervention (mobilization) as a critical modifiable factor.
19	Turan et al. (2023) [19]	Prospective cohort, level II evidence	1,180 elective major surgery patients	Postoperative mobilization measured daily	Minimal mobilization	Composite complications, LOS, and readmissions	Higher early postoperative mobilization associated with reduced composite complications (HR: 0.72) and shorter LOS. Supports systematic mobilization protocols as system-level interventions.
20	de Almeida et al. (2017) [20]	RCT, level II evidence	94 abdominal cancer surgery patients	Early mobilization program (day of surgery)	Standard care	Functional capacity, LOS, and complications	Early mobilization improved functional capacity (6MWT +18%), reduced LOS by ~1.2 days, and lowered pulmonary complications. Confirms rapid postoperative mobilization is a modifiable behavioral factor with measurable recovery benefits.
21	Castelino et al. (2016) [21]	Systematic review, level I evidence	32 studies; 4,800+ patients, abdominal and thoracic surgery	Early mobilization protocols postoperatively	Standard mobilization	Postoperative complications, LOS, pulmonary function, and functional recovery	Early mobilization consistently reduced pulmonary complications, shortened LOS, and improved functional recovery. Reinforces mobilization as a critical modifiable behavioral and system-level factor.
22	Ramesh et al. (2017) [22]	Systematic review and meta-analysis, level I evidence	15 RCTs; 1,200+ cardiac surgery patients	Preoperative patient education programs (cognitive, procedural, and behavioral counseling)	Standard care/no education	Complications, LOS, anxiety, and patient satisfaction	Preoperative education significantly reduced postoperative complications, decreased LOS, lowered patient anxiety, and increased satisfaction. Demonstrates behavioral interventions’ impact on recovery.
23	Klaiber et al. (2018) [23]	Cluster RCT, level II evidence	525 patients; major visceral surgery	Structured preoperative education (PEDUCAT trial)	Standard preoperative instruction	Complications, adherence to perioperative protocols, and LOS	Preoperative education improved adherence to ERAS protocols, reduced specific complications (e.g., infections), and shortened LOS. Highlights the role of system-level and behavioral interventions in recovery optimization.
24	Wick et al. (2017) [24]	Narrative review, level II evidence	Surgical patients across multiple specialties	Postoperative multimodal analgesia (nonopioid pharmacologic + regional techniques)	Opioid-only analgesia	Pain scores, opioid consumption, complications, and LOS	Multimodal analgesia improved pain control, reduced opioid use and related complications, and decreased LOS. Pain management is a modifiable perioperative factor critical for early recovery.
25	Sauro et al. (2024) [25]	Retrospective cohort, level II evidence	12,000+ patients; multiple surgical disciplines	ERAS guideline adherence	Standard perioperative care	LOS, readmissions, complications, and mortality	Full ERAS implementation associated with reduced LOS (~1.5 days), lower readmissions, fewer complications, and no increase in mortality. Confirms ERAS protocols as a system-level modifiable factor that significantly impacts recovery.
26	Ni et al. (2015) [26]	Meta-analysis, level I evidence	10 studies; 850 hepatectomy patients	ERAS programs	Conventional perioperative care	Complications, LOS, and mortality	ERAS programs reduced postoperative complications (OR: 0.62), decreased LOS, and improved overall recovery. Highlights the universal applicability of system-level optimization across surgical types.
27	Desebbe et al. (2016) [27]	Narrative review, level II evidence	Perioperative surgical populations	Perioperative surgical home model (patient-centered, coordinated care)	Standard perioperative care	LOS, costs, patient satisfaction, and complications	Implementation of the perioperative surgical home reduced LOS and costs, improved patient satisfaction, and enhanced protocol adherence. System-level care models are a high-impact modifiable factor for optimizing recovery.
28	Wells et al. (2022) [28]	Scoping review, level II evidence	45 studies; 3,500+ abdominal surgery patients	Wearable devices for continuous postoperative monitoring	Standard in-hospital monitoring	Physiologic metrics, recovery trajectories, and early complication detection	Wearable devices provided real-time monitoring, improved early detection of complications, and enhanced patient engagement. Technology represents a modifiable system-level factor influencing recovery monitoring.
29	Knight et al. (2021) [29]	Systematic review, level I evidence	38 studies; 4,200+ surgical patients	Mobile devices and wearable technologies for patient outcomes	Standard follow-up	Postoperative mobility, complications, adherence, and patient-reported outcomes	Mobile and wearable technologies reliably measured recovery metrics, supported early intervention, and improved patient engagement. Emphasizes system and behavioral innovation in recovery optimization.
30	Muntean et al. (2025) [30]	Systematic review, level I evidence	25 studies; colorectal surgery patients	Continuous wearable-sensor monitoring	Standard monitoring	Complications, predictive analytics, and functional recovery	Continuous wearable monitoring improved early complication detection, informed predictive analytics, and supported personalized recovery plans. Demonstrates technology-driven modifiable system-level interventions.
31	Edney et al. (2024) [31]	Scoping review, level II evidence	30 studies; cardiac surgery patients	Wearable devices to monitor postoperative activity	Standard monitoring	Activity metrics, adherence, and complications	Wearable activity monitoring promoted early mobilization, tracked functional recovery, and facilitated early intervention. Highlights behavioral and system-level modifiable factors.
32	Khan and Shah (2025) [32]	Systematic review, level I evidence	40 studies; general surgical patients	AI-driven wearable sensors for postoperative monitoring	Standard monitoring	Complications, LOS, and functional recovery	AI-integrated wearable sensors enabled continuous remote monitoring, predictive analytics, and early complication alerts. Supports integration of advanced technology as a modifiable system-level intervention to optimize recovery.

A structured risk of bias appraisal was conducted to support the interpretation of the included evidence. Given the heterogeneity of study designs and the narrative nature of this review, formal domain-based tools such as RoB 2 or ROBINS-I were not applied across all studies. Instead, risk of bias was assessed at the level of study design, with randomized controlled trials and systematic reviews/meta-analyses considered to be at lower risk of bias relative to observational studies, and narrative reviews and expert opinion considered to be at higher risk of bias due to their descriptive and non-systematic methodology. This framework was used to guide qualitative synthesis and interpretation of findings rather than to generate numerical quality scores or comparative rankings.

Given the diverse nature of the included studies, spanning multiple surgical procedures, intervention modalities, and outcome measures, quantitative meta-analysis was not feasible. Instead, a qualitative synthesis was performed to identify recurring themes and actionable factors consistently associated with improved postoperative recovery. Studies were categorized into patient, behavioral, and system-level domains, and findings were analyzed to elucidate both direct and indirect influences on recovery trajectories. Using this method, a curated selection of 32 studies was compiled. This qualitative, thematic approach provides an integrated understanding of the multifactorial determinants of postoperative recovery, offering a robust foundation for recommendations and future research directions in general surgery.

Conceptual framework of postoperative recovery

Postoperative recovery is a dynamic, multidimensional process arising from the complex interactions of patient-level physiology, behavioral engagement, and system-level infrastructure. To advance both clinical practice and research, recovery should be conceptualized as a mechanistic, reproducible, and integratable model, in which each domain is defined, measurable, and modifiable, and their interactions are explicitly understood and operationalized.

Patient-Level Determinants

Patient-level factors define the intrinsic physiologic capacity for recovery [6,7,10-16]. Key components include nutritional status, sarcopenia, frailty, comorbidities, metabolic control, and baseline functional reserve [6,7,10-16]. Each factor influences tissue repair, immunologic competence, and susceptibility to complications [6,7,10-16]. To enable reproducibility, these elements should be quantified using validated measures, for example, frailty indices, handgrip strength, muscle mass imaging, albumin/prealbumin levels, or HbA1c for metabolic control [6,7,10-16]. Patient-level determinants form the substrate on which behavioral and system-level interventions act, establishing both the risk profile and the potential ceiling for functional recovery [6,7,10-16].

Behavioral Determinants

Behavioral factors translate physiologic capacity into functional outcomes [17-20]. This includes early mobilization, structured rehabilitation, adherence to prehabilitation programs, patient education, multimodal pain management, and psychological resilience [8-12,17-20,23,25]. To ensure reproducibility and integration, behavioral engagement should be systematically measured and monitored, using objective activity metrics (e.g., step counts and physiologic effort), adherence logs, pain scores, and patient-reported outcome measures (PROMs) [17-20,23-25]. Behavioral determinants are both mediators and modulators: they amplify physiologic reserve, while deficiencies in engagement may blunt potential recovery even in patients with robust baseline function [17-20,23,25].

Despite strong evidence supporting behavioral optimization strategies, real-world implementation is influenced by important clinical and logistical constraints. The feasibility of early mobilization, for example, may be limited by postoperative pain, the presence of epidural analgesia, surgical drains, hemodynamic instability, or admission to intensive care settings, particularly following major abdominal or oncologic procedures. Similarly, adherence to multimodal analgesia and mobilization protocols may vary depending on patient cooperation, delirium risk, and baseline functional status.

The impact and timing of behavioral interventions also differ according to surgical complexity and physiologic reserve. Patients undergoing minimally invasive or elective procedures typically achieve earlier mobilization and faster recovery trajectories compared with those undergoing open, high-risk, or emergency surgery, where postoperative instability and resource requirements may delay implementation of standardized recovery pathways. Likewise, frail, sarcopenic, or cognitively impaired patients may require individualized adaptation of behavioral protocols to ensure safety and feasibility.

These considerations highlight that behavioral determinants of recovery operate within clinical constraints that necessitate tailoring of enhanced recovery strategies to patient condition, operative factors, and institutional resources, rather than uniform application across all surgical populations.

System-Level Determinants

System-level factors provide the organizational scaffolding that ensures consistent, scalable, and adaptive delivery of interventions [1-3,5,21,22]. These include multidisciplinary pathways, perioperative surgical home models, coordinated care protocols, telemonitoring platforms, and digital feedback systems [1-3,5,21,22,26-29]. For reproducibility, these elements should be explicitly defined and standardized, including staffing ratios, pathway components, escalation criteria, and monitoring thresholds [1-3,5,21]. System-level determinants enable fidelity of behavioral and patient-level interventions, and their adaptive feedback mechanisms allow real-time course correction in response to physiologic or behavioral deviations [26-29].

Mechanistic Interactions and Feedback Loops

The interactions among domains are nonlinear, synergistic, and context-dependent. Patient-level vulnerabilities may be mitigated by robust behavioral engagement supported by structured system-level interventions. Conversely, misalignment, such as inadequate monitoring, poor adherence, or unaddressed physiologic deficits, can compound risk and delay recovery. Feedback loops operationalize these interactions: real-time monitoring of physiologic parameters, behavioral adherence, and system-level triggers enables adaptive intervention, creating dynamic correction mechanisms that optimize recovery trajectories [26-30]. Metrics can include continuous vital sign monitoring, wearable activity sensors, PROMs, and AI-driven predictive alerts [26-30].

Contextual Modulators

Contextual factors, including institutional resources, staffing expertise, access to technology, patient comorbidity burden, baseline functional capacity, and sociodemographic factors, shape recovery trajectories. These elements influence both the feasibility and magnitude of intervention effects, and must be explicitly accounted for in both research and clinical implementation to ensure reproducibility and generalizability [1-3,5,21].

Integration and Operationalization

This framework is designed to be integrable across research and clinical applications. For research, it provides a reproducible framework to guide study design, endpoint selection, risk stratification, and intervention targeting. For clinical application, it enables precision perioperative pathways, in which interventions are prioritized based on individual patient risk, monitored for behavioral adherence, and adaptively modulated using system-level infrastructure and feedback loops. Emerging technologies, including wearable sensors, digital health platforms, and AI-based predictive analytics, serve as the backbone of this integration, providing actionable data streams for real-time optimization [26-30].

In sum, the proposed conceptual framework positions postoperative recovery as a mechanistically coherent, measurable, adaptive, and context-sensitive process that bridges patient physiology, behavioral science, and system-level organization. It provides a reproducible blueprint for intervention design, implementation, and evaluation, and establishes a scalable paradigm for precision-guided perioperative care, applicable across heterogeneous surgical populations and healthcare systems.

Patient-level modifiable factors

Patient-level modifiable factors constitute a critical domain of perioperative optimization, encompassing physiologic, metabolic, behavioral, and psychosocial determinants that can be actively addressed prior to surgery [8-12,14-16]. Unlike immutable characteristics such as age or chronic comorbidities, these factors offer opportunities to enhance surgical resilience, reduce complication rates, and improve functional recovery [8-12,14-16]. Emerging evidence demonstrates that integrated prehabilitation programs targeting these domains significantly influence perioperative outcomes, particularly when combined with system-level interventions and structured behavioral strategies [8-12].

Nutritional Status and Sarcopenia

Nutritional status and sarcopenia are among the most influential patient-level determinants of postoperative recovery [11,14-16]. Malnutrition, defined by inadequate protein-energy intake or micronutrient deficiencies, disrupts collagen deposition, reduces immune competence, and impairs wound healing [11,14]. Mechanistically, protein-energy deficits compromise fibroblast proliferation and collagen crosslinking, while deficiencies in vitamins such as C and D, zinc, and selenium attenuate antioxidant defenses and immune cell function [9,14]. These disruptions increase the risk of surgical site infections, anastomotic leaks, and delayed mobilization [14-16].

Sarcopenia, characterized by the progressive loss of skeletal muscle mass and functional strength, often coexists with malnutrition and independently predicts adverse surgical outcomes [15,16]. Reduced muscle mass limits physiologic reserve, decreases tolerance to surgical stress, and impairs engagement in postoperative rehabilitation [15-17]. Cross-sectional imaging, bioimpedance analysis, and functional measures, such as handgrip strength, help identify high-risk patients [14-16]. Multimodal interventions integrating protein supplementation, resistance training, and functional mobility exercises have been shown to improve postoperative strength, enhance functional recovery, and reduce complication rates [8,9,11]. Evidence from colorectal and abdominal surgery cohorts indicates that even short-term nutritional and exercise interventions can meaningfully enhance postoperative functional capacity and reduce length of stay, highlighting the modifiable nature of these risk factors [8,9,11].

Frailty and Physical Conditioning

Frailty is a multidimensional syndrome reflecting reduced physiologic reserve and impaired ability to withstand surgical stress [6,7,13]. It encompasses deficits in musculoskeletal strength, cardiovascular and respiratory function, and functional independence [6,7,13]. Mechanistically, frailty is associated with impaired inflammatory regulation, diminished mitochondrial function, and reduced adaptive response to stress, increasing susceptibility to postoperative complications, including respiratory failure, delirium, and prolonged hospitalization [7,13].

Frailty indices, integrating measures of mobility, strength, energy, and cognition, provide robust prognostic tools that surpass chronological age in predicting postoperative risk [12,13]. Prehabilitation programs targeting frailty utilize structured aerobic conditioning, resistance exercise, inspiratory muscle training, and functional mobility regimens [6-7,10]. Clinical studies demonstrate that such interventions increase physiologic reserve, enhance tolerance to anesthesia and surgical stress, and facilitate early mobilization, ultimately reducing complication rates and improving discharge readiness [6,7,12]. The individualized tailoring of these programs based on baseline frailty, comorbidities, and anticipated surgical stress is essential to optimize outcomes and ensure meaningful functional improvement [6,7,10].

Glycemic Control and Metabolic Optimization

Metabolic health, particularly glycemic control, plays a central role in postoperative outcomes [14]. Hyperglycemia impairs neutrophil chemotaxis and phagocytosis, induces endothelial dysfunction, and exacerbates oxidative stress, leading to increased rates of surgical site infection, delayed wound healing, and anastomotic complications [14]. Insulin resistance and metabolic syndrome amplify these risks by promoting chronic low-grade inflammation and impairing tissue repair mechanisms [14].

Perioperative glycemic optimization, through preoperative assessment, tight intraoperative control, and postoperative monitoring, reduces infectious complications and improves functional recovery [14]. Broader metabolic interventions, including weight management, structured exercise, and dietary modulation, improve insulin sensitivity, enhance immune competence, and support physiologic resilience [8,9,14]. Targeted approaches for patients with obesity or diabetes are particularly impactful, demonstrating reduced morbidity and accelerated return to baseline activity [6,7,14]. The integration of metabolic optimization into prehabilitation protocols represents a critical step toward individualized, risk-adjusted perioperative care [8,9,14].

Tobacco and Alcohol Use

Tobacco and alcohol consumption are well-established modifiable risk factors with profound impacts on surgical recovery [8,32]. Tobacco exposure impairs endothelial function, reduces tissue oxygenation, increases oxidative stress, and delays wound healing, contributing to pulmonary complications, anastomotic failure, and increased infection rates [8,32]. Alcohol misuse similarly disrupts hepatic metabolism, impairs immune function, and increases the risk of coagulopathy and postoperative bleeding [8,32].

Preoperative cessation programs, even when initiated several weeks before surgery, significantly mitigate these risks [8,32]. Smoking cessation enhances tissue perfusion and reduces pulmonary complications, while alcohol reduction improves hepatic and immune function and decreases susceptibility to infection [8,32]. Structured cessation programs, behavioral counseling, and pharmacologic adjuncts integrated into preoperative pathways optimize adherence and maximize physiologic recovery [8,32]. The impact of these interventions demonstrates that behavioral modifications can produce clinically meaningful improvements in surgical outcomes when systematically applied [8,32].

Sleep, Psychological Health, and Stress

Sleep quality, psychological health, and stress are critical yet frequently underrecognized determinants of recovery [32]. Sleep disruption impairs immune function, dysregulates hormonal stress responses, and alters pain perception, negatively affecting postoperative mobility and functional recovery [32]. Psychological distress, including anxiety and depression, diminishes engagement with rehabilitation protocols, reduces adherence to perioperative instructions, and is associated with increased complications and prolonged hospitalization [32].

Interventions targeting these factors (Table 2), such as cognitive behavioral therapy, structured patient education, mindfulness techniques, and sleep hygiene optimization, have demonstrated improvements in both objective and patient-reported recovery outcomes [24,25,32]. Optimizing psychological health enhances adherence to multimodal recovery protocols, supports early mobilization, and facilitates engagement in prehabilitation exercises [24,25,32]. Recognizing the interdependent effects of stress, sleep, and psychological well-being allows for a comprehensive, patient-centered approach that addresses the full spectrum of modifiable patient-level determinants of postoperative recovery [24,25,32].

**Table 2:** Patient-Level Modifiable Factors Affecting Postoperative Recovery Summary of patient-level modifiable factors influencing postoperative recovery, including mechanisms, assessment methods, interventions, and clinical outcomes. Factors include nutrition, sarcopenia, frailty, metabolic control, substance use, sleep, and psychological health, highlighting evidence-based strategies to optimize surgical resilience and recovery [1-11,13-18,20-26,30-32]. BMI: body mass index, CT: computed tomography, MRI: magnetic resonance imaging, CBT-I: cognitive behavioral therapy for insomnia, PHQ-9: Patient Health Questionnaire-9, GAD-7: Generalized Anxiety Disorder-7

Factor	Mechanistic impact on recovery	Assessment/identification	Evidence-based interventions	Reported outcomes/clinical impact
Nutritional status/malnutrition	Impairs collagen deposition, fibroblast proliferation, and immune competence; delays wound healing; increases infection and anastomotic leak risk	Serum albumin/prealbumin, BMI, and dietary assessment	Protein-energy supplementation, micronutrient optimization (vitamins C/D, zinc, and selenium), and dietitian-led counseling	Reduced surgical site infections, improved wound healing, enhanced functional recovery, and decreased length of stay
Sarcopenia/muscle mass deficiency	Decreased physiologic reserve, limited stress tolerance, and impaired postoperative mobility	Cross-sectional imaging (CT/MRI), bioimpedance, handgrip strength, and gait speed	Resistance training, functional mobility exercises, protein supplementation, and multimodal prehabilitation	Increased postoperative strength, faster functional recovery, lower complication rates, and shorter hospitalization
Frailty	Reduced physiologic reserve, impaired inflammatory and mitochondrial function, and higher susceptibility to complications	Frailty indices (mobility, strength, energy, and cognition) and clinical scoring systems	Tailored aerobic and resistance exercise, inspiratory muscle training, and functional mobility programs	Reduced postoperative complications (delirium and respiratory failure), improved discharge readiness, and better tolerance to surgical stress
Glycemic control/metabolic optimization	Hyperglycemia impairs neutrophil function, endothelial integrity, and wound healing; insulin resistance promotes chronic inflammation	HbA1c, fasting glucose, and insulin resistance indices	Preoperative optimization, perioperative insulin management, structured exercise, and dietary modulation	Reduced surgical site infections, improved wound healing, faster return to baseline activity, and lower morbidity in diabetic/obese patients
Tobacco use	Endothelial dysfunction, reduced tissue oxygenation, oxidative stress, delayed wound healing, and increased pulmonary complications	Smoking history and cotinine testing	Preoperative cessation programs, behavioral counseling, and pharmacologic aids (nicotine replacement and varenicline)	Reduced pulmonary complications, improved wound healing, and lower infection and anastomotic leak rates
Alcohol use	Hepatic dysfunction, coagulopathy, impaired immune response, and increased bleeding risk	Alcohol consumption history and AUDIT-C screening	Preoperative reduction/cessation programs, counseling, and structured support	Decreased postoperative bleeding, improved immune competence, and reduced infectious complications
Sleep disturbance/circadian disruption	Impaired immune function, hormonal dysregulation, altered pain perception, and delayed mobilization	Sleep questionnaires, actigraphy, and patient-reported sleep quality	Sleep hygiene optimization, CBT-I, and environmental modifications	Enhanced immune function, reduced pain perception, and improved mobilization and functional recovery
Psychological stress/mental health	Anxiety, depression reduce rehabilitation adherence, dysregulate stress responses, and increase complications	Standardized scales (PHQ-9 and GAD-7) and clinical interview	Cognitive behavioral therapy, mindfulness, structured patient education, and stress reduction programs	Improved adherence to prehabilitation and recovery protocols, reduced complication rates, shorter length of stay, and enhanced patient-reported outcomes

Behavioral determinants of recovery

Behavioral factors exert a profound influence on postoperative recovery, modulating both physiologic and functional outcomes across diverse surgical populations [8-12,14,32]. These determinants include early mobilization, pain management, patient engagement, and perioperative education, each of which interacts with patient-level modifiable factors such as nutritional status, sarcopenia, frailty, and metabolic health [8-12,14,32]. Behavioral optimization not only directly improves recovery but also enhances adherence to prehabilitation and Enhanced Recovery After Surgery (ERAS) protocols, amplifying the benefits of structured perioperative pathways [8-12,14]. High-quality evidence indicates that these interventions can reduce complications, accelerate functional recovery, and improve both objective and patient-reported outcomes, particularly in high-risk surgical cohorts [8-12,14].

Early Mobilization

Early postoperative mobilization is consistently identified as a cornerstone of accelerated recovery, with mechanistic effects spanning multiple physiologic systems [8,9,12]. Immobility induces rapid skeletal muscle atrophy, endothelial dysfunction, venous stasis, insulin resistance, and pulmonary compromise [8,9,12]. Early mobilization mitigates these processes by enhancing microvascular perfusion, promoting venous return, stimulating gastrointestinal motility, and supporting diaphragmatic excursion and pulmonary function [8,9,12]. Randomized controlled trials demonstrate that mobilization within 24-48 hours after major abdominal, colorectal, and oncologic surgery reduces pulmonary complications, thromboembolic events, and postoperative ileus while accelerating the restoration of functional independence [8,9,12]. Structured protocols with progressive ambulation and supervised physical therapy improve step count, respiratory mechanics, and muscle strength postoperatively, even in frail or sarcopenic patients [8,9,12]. Early mobilization synergizes with nutritional and metabolic optimization, enabling patients to translate preserved physiologic reserve into meaningful functional gains, thereby shortening hospital length of stay and reducing post-discharge complications [8,9,12].

Pain Management and Multimodal Analgesia

Pain is a critical behavioral determinant that directly impacts functional recovery, mobilization, and overall physiologic stress [8,10,32]. Inadequate analgesia activates the sympathetic nervous system, elevating cortisol and catecholamines, which exacerbate hyperglycemia, impair immune function, and interfere with wound healing [8,10,32]. It also diminishes engagement with mobilization and rehabilitation protocols, prolonging recovery [8,10,32]. Multimodal analgesia, integrating regional anesthesia, non-opioid analgesics, acetaminophen, nonsteroidal anti-inflammatory drugs (NSAIDs), gabapentinoids, and adjunctive modalities, provides superior analgesia while minimizing opioid-related side effects [8,10,32]. Evidence demonstrates that patients managed with multimodal pain strategies mobilize earlier, achieve higher functional scores at discharge, experience fewer complications such as pulmonary events and ileus, and have reduced readmission rates [8,10,32]. Individualized pain regimens tailored to surgical complexity, patient comorbidities, and preoperative opioid exposure maximize recovery while minimizing physiologic and behavioral barriers imposed by uncontrolled pain [8,10,32].

Patient Engagement and Adherence

Patient engagement is a critical mediator of behavioral recovery, influencing adherence to mobilization, nutrition, medication, and rehabilitation protocols [8,9,12,14,32]. Mechanistically, engaged patients demonstrate improved self-efficacy, motivation, and adherence to prescribed behaviors, which reinforce physiologic and functional recovery [8,9,12]. Structured engagement programs, including motivational interviewing, personalized goal-setting, and digital health interventions, significantly improve compliance with ERAS protocols and early mobilization schedules [8,9,12]. Engagement strategies are particularly important for high-risk or frail patients, as they mitigate the negative impact of baseline physiologic vulnerabilities on postoperative outcomes [8,9,12]. Patients who actively participate in their care consistently demonstrate faster return to baseline functional capacity, reduced complication rates, and higher satisfaction with care [8,9,12]. Fostering engagement is therefore an integral determinant of perioperative success [8,9,12].

Perioperative Education

Structured perioperative education enhances recovery by improving patient knowledge, reducing anxiety, and promoting adherence to recovery protocols [8,24,25,32]. Education modulates behavioral and physiologic responses to surgical stress, leading to reduced perioperative cortisol levels, improved immune function, and enhanced pain-coping mechanisms [24,25,32]. Comprehensive programs that include preoperative counseling, multimedia materials, and post-discharge reinforcement are associated with decreased complication rates, shorter hospital stays, and improved patient-reported outcomes [24,25,32]. Education also facilitates integration of behavioral and patient-level interventions, enabling patients to participate more effectively in mobilization, nutritional optimization, and self-monitoring strategies [24,25,32]. By creating a foundation of informed engagement, perioperative education ensures that behavioral interventions achieve maximal impact across diverse surgical populations [24,25,32].

Behavioral determinants of recovery (Table 3), comprising early mobilization, multimodal pain management, patient engagement, and structured education, interact dynamically with patient-level physiologic factors to shape postoperative outcomes [8-12,14,24,25,32]. These interventions influence both objective measures such as complication rates, functional independence, and length of stay, as well as subjective patient-reported outcomes, including pain, readiness for discharge, and overall satisfaction [8-12,14,24,25,32]. Recognizing and strategically optimizing behavioral determinants allows perioperative care teams to enhance the efficacy of patient-level and system-level interventions, ultimately creating an integrated, patient-centered approach to surgical recovery that maximizes functional resilience, minimizes complications, and accelerates return to baseline health [8-12,14,24,25,32].

**Table 3:** Summary of Behavioral Determinants of Postoperative Recovery This table summarizes behavioral determinants of postoperative recovery, integrating physiologic mechanisms, patient-level interactions, intervention strategies, and observed clinical outcomes. Behavioral optimization directly influences functional recovery, complication rates, and adherence to perioperative protocols, particularly in patients with modifiable risk factors such as frailty, sarcopenia, and metabolic dysregulation [8-12,14,24,25,32]. NSAIDs: nonsteroidal anti-inflammatory drugs

Behavioral factor	Physiologic/mechanistic rationale	Patient-level interaction	Intervention/strategy	Observed outcomes
Early mobilization	Reduces skeletal muscle atrophy, venous stasis, pulmonary compromise, and insulin resistance, and enhances microvascular perfusion, gastrointestinal motility, and diaphragmatic excursion	Frail or sarcopenic patients have reduced baseline physiologic reserve; early ambulation mitigates risk of deconditioning and functional decline	Structured ambulation protocols, supervised physical therapy, and progressive mobilization within 24-48 hours postoperatively	Reduced pulmonary and thromboembolic complications, decreased postoperative ileus, accelerated functional independence, shorter hospital length of stay, and lower incidence of post-discharge complications
Pain management/multimodal analgesia	Inadequate analgesia activates sympathetic response, elevates cortisol and catecholamines, impairs immune function, and delays wound healing	Poorly controlled pain limits engagement in mobilization and rehabilitation; may exacerbate hyperglycemia and metabolic stress	Multimodal analgesia incorporating regional anesthesia, NSAIDs, acetaminophen, gabapentinoids, and adjunctive techniques; opioid-sparing regimens tailored to patient comorbidities	Earlier mobilization, higher functional scores at discharge, reduced pulmonary and ileus complications, and decreased readmissions
Patient engagement/adherence	Engagement improves self-efficacy, motivation, and adherence to recovery protocols, reinforcing physiologic and functional recovery	High-risk or frail patients benefit most; adherence mediates the effectiveness of nutrition, mobility, and metabolic optimization interventions	Motivational interviewing, personalized goal-setting, digital health tools, and structured follow-up and reinforcement	Faster return to baseline functional capacity, reduced complication rates, and improved patient satisfaction and adherence to multimodal recovery protocols
Perioperative education	Reduces perioperative stress, lowers cortisol, improves immune response, and enhances coping with pain and rehabilitation	Supports adherence to early mobilization, nutritional optimization, and multimodal strategies, and improves patient understanding of individualized risk factors	Preoperative counseling, multimedia educational materials, post-discharge reinforcement, and group or individualized sessions	Decreased complication rates, shorter hospital stay, improved patient-reported outcomes, and enhanced adherence to behavioral and recovery interventions

System-level determinants

System-level determinants encompass the organizational, infrastructural, and procedural frameworks that govern the delivery of perioperative care [8-12,14,24,32]. While patient-level and behavioral factors shape recovery at the individual level, system-level interventions define the environment in which these processes operate, influencing the consistency, efficiency, and effectiveness of care delivery [8-12,24,32]. System-level factors include multidisciplinary perioperative pathways, coordinated perioperative models such as the perioperative surgical home, telemonitoring and digital health platforms, and structured implementation strategies grounded in evidence-based principles [8-12,14,24,32]. By standardizing care, reducing variability, and optimizing resource allocation, these interventions lay a foundation for patient-level and behavioral optimizations that reliably translate into improved postoperative outcomes [8-12,14,24,32]. High-functioning perioperative systems reduce complications, enhance functional recovery, minimize hospital length of stay, and improve both patient-reported outcomes and institutional efficiency, particularly in high-risk or complex surgical populations [8-12,14,24,32].

Multidisciplinary Perioperative Care Pathways

Multidisciplinary care pathways, exemplified by Enhanced Recovery After Surgery (ERAS) protocols, integrate surgical, anesthetic, nursing, physiotherapy, nutritional, and pharmacy expertise into structured perioperative plans [8-12,14]. Mechanistically, these pathways ensure timely mobilization, standardized analgesia, optimized fluid and metabolic management, and proactive nutritional support [8-12,14]. By establishing clear benchmarks for each perioperative phase, pathways reduce variability in care delivery and mitigate errors associated with fragmented management [8-12,14]. Meta-analytic evidence indicates that institutions implementing multidisciplinary pathways experience reductions in postoperative morbidity, including pulmonary complications, surgical site infections, venous thromboembolism, and ileus, while concurrently decreasing hospital length of stay and readmission rates [8-12,14]. Such pathways provide the structural framework necessary to support patient-level interventions such as prehabilitation, glycemic optimization, and nutritional supplementation, highlighting the interdependence of system-level and individual-level determinants [8-12,14]. The degree of pathway success is closely linked to institutional culture, staff engagement, and adherence monitoring, illustrating that effective system-level design requires both structural and behavioral alignment [8-12,14].

Surgical Home and Coordinated Perioperative Models

The perioperative surgical home represents an advanced model of coordinated, patient-centered care that encompasses preoperative risk stratification, intraoperative optimization, and post-discharge follow-up [8,12,14,32]. Mechanistically, this model reduces fragmentation, anticipates complications, and ensures continuity across multiple care settings [8,12,14,32]. Structured preoperative assessments identify physiologic vulnerabilities, psychosocial factors, and comorbidities that may affect recovery, enabling targeted interventions before surgery [8,12,14,32]. Coordinated care pathways facilitate real-time communication among providers, accelerate decision-making in response to complications, and standardize rehabilitation milestones [8,12,14,32]. Empirical data indicate that hospitals adopting surgical home models achieve significant reductions in postoperative morbidity, unplanned ICU admissions, and hospital length of stay, while improving patient satisfaction and adherence to recovery protocols [8,12,14,32]. The integration of centralized care coordination and multidisciplinary case conferences further enhances the capacity to deliver individualized, patient-centered interventions that amplify the efficacy of behavioral and physiologic optimizations [8,12,14,32].

Telemonitoring and Digital Perioperative Platforms

Telemonitoring and digital health platforms represent an emerging approach to extending perioperative monitoring beyond the inpatient setting [8,12,14,24,25]. These systems utilize wearable sensors, mobile applications, and remote monitoring tools to track physiologic parameters, mobility, pain scores, wound-related indicators, and patient-reported outcomes during the postoperative period [8,12,14,24,25]. In early-phase studies, these platforms have been associated with improved detection of postoperative deviations from expected recovery trajectories and may facilitate earlier clinical intervention [8,12,14,24,25]. However, the current evidence base is predominantly derived from pilot studies, feasibility trials, and observational cohorts, with limited high-quality randomized data evaluating their impact on hard clinical outcomes.

Preliminary findings suggest potential benefits in reducing readmissions, improving adherence to postoperative recovery pathways, and enhancing patient engagement through feedback loops, reminders, and educational reinforcement [8,12,14,24,25]. Emerging applications of predictive analytics and machine learning algorithms may further enhance risk stratification and early complication detection, although these approaches remain in early stages of clinical validation [8,12,14,24,25]. Overall, digital perioperative platforms should currently be viewed as adjunctive tools with promising but evolving evidence, rather than established components of standard perioperative care.

Implementation Science in ERAS Programs

Implementation science principles are critical to ensuring that evidence-based perioperative interventions achieve maximal impact [8-12,14,24]. Successful adoption of enhanced recovery programs requires structured workflow integration, staff education, audit and feedback mechanisms, and continuous quality improvement initiatives [8-12,14,24]. Mechanistically, structured implementation minimizes adherence variability, reinforces fidelity to multimodal recovery protocols, and ensures the sustainability of programmatic gains [8-12,14,24]. Institutions applying formal implementation frameworks achieve higher protocol adherence, reduced postoperative morbidity, shorter hospital stays, and improved functional outcomes [8-12,14,24]. Implementation science also addresses barriers such as staff resistance, logistical limitations, and resource constraints, thereby allowing patient-level and behavioral interventions to operate within a robust, supportive environment [8-12,14,24]. By coupling structured implementation with continuous monitoring and feedback, system-level strategies ensure that the full spectrum of perioperative optimizations, physiologic, behavioral, and organizational, is consistently applied to all patients [8-12,14,24].

System-level determinants (Table 4) provide the structural and organizational scaffolding necessary for high-quality perioperative care. Multidisciplinary care pathways, surgical home models, telemonitoring, and implementation science frameworks reduce variability, standardize evidence-based interventions, and facilitate early identification and management of complications [8-12,14,24,25,32]. These interventions amplify the impact of patient-level and behavioral optimizations, ensuring that physiologic resilience, mobilization, pain management, and education translate into tangible improvements in recovery [8-12,14,24,25,32]. Recognizing the central role of system-level determinants is essential for designing integrated, patient-centered perioperative programs that optimize both short-term and long-term outcomes, particularly in complex or high-risk surgical populations [8-12,14,24,25,32]. High-functioning systems not only improve clinical outcomes but also enhance institutional efficiency, patient satisfaction, and long-term functional recovery, underscoring their critical role in contemporary surgical practice [8-12,14,24,25,32].

**Table 4:** System-Level Determinants of Postoperative Recovery This table summarizes system-level determinants influencing postoperative recovery, including multidisciplinary care pathways, coordinated perioperative models, digital health platforms, and implementation strategies. It integrates underlying mechanisms, interactions with patient-level and behavioral factors, and associated clinical outcomes, highlighting the role of structured systems in reducing variability, improving adherence, and optimizing recovery trajectories [8-12,14,24,25,32]. ERAS: Enhanced Recovery After Surgery, ICU: intensive care unit

System-level factor	Mechanistic rationale	Patient-level/behavioral interactions	Observed clinical outcomes
Multidisciplinary perioperative care pathways (e.g., ERAS)	Integrates surgical, anesthetic, nursing, physiotherapy, nutrition, and pharmacy care into standardized protocols; ensures timely mobilization, fluid/metabolic optimization, and analgesia; and reduces variability and error from fragmented care	Provides structural support for patient-level optimizations, such as prehabilitation, glycemic control, nutrition, and mobilization, and aligns staff behavior with recovery goals	Reduced postoperative morbidity (pulmonary complications, infections, thromboembolism, and ileus), shorter hospital length of stay, lower readmission rates, and enhanced functional recovery
Perioperative surgical home/coordinated care models	Centralizes care coordination across pre-, intra-, and postoperative phases; anticipates complications; and standardizes assessments, decision-making, and rehabilitation milestones	Enhances patient-level interventions via risk stratification and targeted preoperative optimization, and promotes adherence to mobilization, education, and pain management protocols	Reduced morbidity, fewer unplanned ICU admissions, decreased hospital length of stay, higher patient satisfaction, and improved adherence to recovery protocols
Telemonitoring and digital health platforms	Continuous tracking of physiologic parameters, mobility, pain, wound healing, and patient-reported outcomes, and enables real-time adjustments; predictive analytics anticipate complications	Reinforces patient engagement, adherence to mobilization, nutrition, and multimodal pain strategies, and supports behavior change through feedback loops and educational prompts	Accelerated recovery milestones, fewer readmissions, improved functional and patient-reported outcomes, earlier detection of complications, and personalized care adjustments
Implementation science in ERAS/system programs	Structured integration of workflows, staff education, audit and feedback, and quality improvement; reduces adherence variability; reinforces protocol fidelity; and addresses barriers to adoption	Ensures that patient-level and behavioral interventions (nutrition, frailty mitigation, early mobilization, and education) are consistently applied, and aligns staff practice with evidence-based recovery pathways	Higher protocol adherence, reduced postoperative morbidity, shorter hospital stays, improved functional recovery, and sustained programmatic gains

Interaction between domains

Surgical recovery is best conceptualized as a dynamic, multidimensional process arising from the continuous interplay of patient-level, behavioral, and system-level determinants [8-12,14,24,25,32]. These domains do not operate in isolation; their influence is contingent on context, individual risk profiles, and institutional capabilities, creating a network of interactions in which the efficacy of any single intervention depends on the integrity and alignment of the others. Understanding these interdependencies is essential for designing perioperative programs that are both effective and resilient across diverse patient populations [8-12,14,24,25,32].

Patient-level determinants, including frailty, sarcopenia, nutritional status, and metabolic control, establish the physiologic substrate for recovery [8-12,14,32]. These factors define baseline resilience, tissue repair capacity, immune competence, and susceptibility to complications. However, the expression of physiologic reserve is context-dependent: without supportive behavioral interventions such as early mobilization, structured rehabilitation, and multimodal analgesia, intrinsic physiologic advantages may not translate into improved outcomes [8-12,14,32]. Conversely, well-designed behavioral strategies can partially compensate for vulnerabilities, enabling meaningful functional recovery even among high-risk or frail patients. For instance, targeted prehabilitation combined with pain optimization can amplify muscle strength and cardiopulmonary conditioning, mitigating the adverse impact of sarcopenia on postoperative morbidity [8-12,14,32].

System-level determinants function as the organizational and structural scaffolding that allows patient-level and behavioral interventions to operate reliably [8-12,14,24,25,32]. Multidisciplinary care pathways, perioperative surgical home models, and rigorous implementation frameworks standardize care, minimize variability, and ensure timely escalation of complications [8-12,14,24,25,32]. These systems actively reinforce behavioral engagement: adherence to mobilization schedules, nutritional regimens, and rehabilitation plans improves when supported by institutional protocols, feedback mechanisms, and coordinated monitoring [8-12,14,24,25,32]. Digital health platforms, telemonitoring, and predictive analytics further extend the system's influence by providing real-time physiologic and behavioral data, enabling individualized adjustments, and facilitating early detection of deviations from expected recovery trajectories [8-12,14,24,25,32]. In this sense, system-level infrastructure does more than enforce compliance; it creates an adaptive, feedback-driven environment that maximizes patient physiology and behavioral engagement.

The interactions among these domains are often nonlinear and synergistic. In patients with high physiologic risk or multiple comorbidities, coordinated system-level support magnifies the efficacy of behavioral interventions, optimizing the functional utilization of physiologic reserve [8-12,14,32]. Conversely, misalignment, such as inadequate system support, low patient engagement, or unaddressed physiologic vulnerabilities, can amplify risks, illustrating that deficiencies in one domain may cascade across the network [8-12,14,32]. Contextual factors such as institutional resources, staffing expertise, and care culture further modulate these interactions, explaining variability in outcomes across centers despite similar patient populations and protocols [8-12,14,24,25,32].

Clinically, this multidimensional framework provides a rationale for targeted intervention prioritization. High-risk patients, particularly those with frailty, sarcopenia, or metabolic derangements, benefit most from intensive prehabilitation, structured mobilization, and system-level supports such as telemonitoring and coordinated multidisciplinary oversight [8-12,14,32]. Patients with greater physiologic reserve may require less intensive behavioral or systemic interventions, allowing resources to be allocated efficiently. Integration of emerging technologies, including wearable devices, continuous digital monitoring, and predictive analytics, enables dynamic, individualized adjustment of care, aligning interventions with real-time physiologic and behavioral data [8-12,14,24,25,32]. This approach enhances functional recovery, reduces complications, and increases resilience against unexpected perioperative stressors.

By framing recovery as an orchestrated interaction among interdependent domains, clinicians and health systems can move beyond simplistic, single-domain interventions. Optimizing surgical recovery requires a holistic, evidence-informed strategy that simultaneously addresses physiologic, behavioral, and systemic determinants, dynamically monitors them, and contextually adapts them [8-12,14,24,25,32]. This perspective advances perioperative care from reactive management toward a precision-oriented, patient-centered, and systemically integrated paradigm, with the potential to improve both short-term and long-term functional outcomes across heterogeneous surgical populations [8-12,14,24,25,32].

Current limitations

Despite substantial advances in understanding postoperative recovery, several limitations constrain the interpretation and generalizability of existing evidence. First, much of the current literature is dominated by single-center studies, observational cohorts, or studies with heterogeneous patient populations, limiting the ability to draw causal inferences. Variability in patient selection, comorbidity burden, and baseline functional status introduces significant confounding, particularly when examining the impact of patient-level interventions such as prehabilitation, nutritional optimization, or frailty mitigation. While meta-analyses suggest consistent benefits of structured programs, residual bias, differences in outcome definitions, and inconsistent adherence reporting limit the precision of effect estimates.

Furthermore, much of the existing ERAS and perioperative optimization literature originates from high-volume academic centers with dedicated multidisciplinary teams and structured implementation pathways. As a result, the observed outcomes may not be fully generalizable to lower-resource settings or institutions without dedicated ERAS infrastructure, standardized protocols, or specialized perioperative care teams. Differences in staffing, protocol adherence, and institutional capacity may limit the external validity of reported effect sizes when translated into broader clinical practice.

Second, behavioral determinants of recovery remain challenging to quantify and standardize. Metrics such as patient engagement, adherence to mobilization protocols, or pain management compliance are often reported inconsistently, relying on self-reported measures or surrogate markers. This introduces measurement bias and limits cross-study comparisons. Moreover, the effectiveness of behavioral interventions is highly context-dependent, influenced by staff engagement, institutional culture, and resource availability. Nevertheless, many studies fail to account for these systemic moderating factors, leading to uncertainty about generalizability across diverse clinical settings.

Third, system-level interventions, while increasingly recognized as critical, are heterogeneous in design, implementation fidelity, and reporting. Variability in multidisciplinary pathway composition, perioperative home models, telemonitoring adoption, and digital platform integration complicates interpretation. Additionally, most studies have limited longitudinal follow-up, making it difficult to assess long-term functional recovery, patient-reported outcomes, and cost-effectiveness. The impact of institutional resources, staffing models, and local care culture further modulates outcomes, yet these contextual factors are infrequently quantified, limiting the ability to translate findings across settings.

Finally, integration of emerging technologies such as predictive analytics, AI-driven monitoring, and wearable devices remains in its infancy. While promising, the evidence is largely limited to pilot studies or feasibility trials, and robust, multicenter validation is lacking. Ethical, logistical, and regulatory considerations, as well as disparities in technology access, introduce additional constraints on implementation and widespread adoption.

In addition, publication bias may have influenced the available evidence, as studies reporting positive outcomes following ERAS implementation, prehabilitation, and related perioperative interventions are more likely to be published than studies demonstrating neutral or negative findings. This may lead to an overestimation of the magnitude of benefit in meta-analytic and narrative syntheses.

Collectively, these limitations highlight that while patient-level, behavioral, and system-level interventions show considerable promise, interpretation of their efficacy must be nuanced. Future research should prioritize multicenter, controlled studies with standardized outcome measures, rigorous assessment of adherence and engagement, longitudinal follow-up, and integration of contextual and technological factors. Addressing these gaps is essential for translating current insights into reproducible, high-quality perioperative care that is both patient-centered and scalable across diverse healthcare environments.

Future directions

As perioperative care continues to evolve, future research and clinical innovation must adopt a multidimensional framework that recognizes the interdependence of patient-level, behavioral, and system-level determinants of recovery. At the patient level, deeper investigation is needed into personalized physiologic optimization strategies, including precision nutrition, sarcopenia-targeted interventions, and individualized prehabilitation protocols that account for comorbidities, frailty, and baseline functional reserve. Longitudinal studies are essential to understand how these interventions influence not only early postoperative outcomes but also long-term functional recovery, quality of life, and durable physiologic resilience. Furthermore, robust stratification tools that integrate biomarkers, physiologic indices, and genetic or metabolic profiles could enable targeted interventions for high-risk populations, maximizing efficiency and clinical impact.

Behavioral determinants remain a critical avenue for innovation. Future work should focus on standardizing metrics for engagement, adherence, and rehabilitation participation to enable more reliable cross-study comparisons and meta-analytic synthesis. Integrating behavioral science frameworks to enhance motivation, compliance, and coping strategies, alongside structured pain and symptom management, may amplify the benefits of prehabilitation and postoperative mobilization. Moreover, adaptive behavioral interventions that respond in real time to patient performance and physiologic feedback represent a promising frontier for precision behavioral medicine in surgical care.

System-level determinants will increasingly define the ceiling for achievable recovery outcomes. Implementation science should explore scalable, high-fidelity perioperative pathways that integrate multidisciplinary coordination, telemonitoring, and dynamic resource allocation to support individualized care. Emerging digital health technologies, including wearable sensors, AI-driven predictive analytics, and continuous physiologic monitoring, offer unprecedented opportunities to detect early deviations from expected recovery trajectories, personalize interventions, and optimize outcomes in real time. Validation of these technologies across multicenter, heterogeneous populations, coupled with attention to ethical, regulatory, and equity considerations, will be critical for translating innovation into routine clinical practice.

Finally, the future of perioperative care lies in holistic, integrated, and adaptive strategies that combine physiologic optimization, behavioral engagement, and system-level orchestration. Research should move beyond siloed interventions toward dynamic, feedback-driven care models that anticipate patient vulnerabilities, adapt interventions in real time, and continuously evaluate outcomes. Such a paradigm has the potential to improve short-term recovery, reduce complications, enhance functional independence, and support patient-centered, sustainable surgical care at scale. By focusing on integration, personalization, and technological innovation, future directions in perioperative recovery can transform the trajectory of surgical outcomes across diverse patient populations and healthcare systems.

## Conclusions

Postoperative recovery is a complex, multidimensional process that arises from the dynamic interplay among patient-level physiology, behavioral engagement, and system-level infrastructure. Optimal recovery is achieved not solely through surgical technique, but through the coordinated mitigation of physiologic vulnerabilities such as frailty, sarcopenia, and metabolic dysregulation; the promotion of active behavioral participation through early mobilization, structured rehabilitation, and patient-centered education; and the implementation of robust system-level frameworks that ensure fidelity, adaptability, and real-time monitoring. The interdependencies among these domains are nonlinear and context-sensitive, with synergistic feedback loops that amplify or attenuate recovery trajectories. Patient-level deficits can be offset by targeted behavioral interventions supported by structured care pathways, whereas misalignment across any domain can exponentially increase risk and prolong recovery. Emerging digital health technologies, wearable sensors, and predictive analytics provide unprecedented opportunities to quantify recovery, anticipate complications, and tailor interventions with precision, advancing perioperative care beyond traditional paradigms. Conceptualizing postoperative recovery as an integrated, reproducible, and mechanistically coherent framework enables clinicians and health systems to shift from reactive management to precision-guided, patient-centered strategies. By harmonizing physiologic optimization, behavioral engagement, and system-level orchestration, perioperative care can achieve more consistent, resilient, and measurable outcomes. This integrated approach establishes a blueprint for improving functional recovery, reducing complications, and enhancing the overall patient experience across diverse surgical populations, setting the stage for the next generation of evidence-informed, high-value perioperative care.
